# Association Between Selected Flavanols and Isoflavones and Precocious Puberty in Girls—A Scoping Review

**DOI:** 10.3390/nu18060879

**Published:** 2026-03-10

**Authors:** Izabela Michońska, Agata Serwin, Katarzyna Dereń

**Affiliations:** 1 Faculty of Health Sciences and Psychology, Collegium Medicum, University of Rzeszów, 35-959 Rzeszów, Poland; kderen@ur.edu.pl; 2 Independent Researcher, 05-070 Sulejówek, Poland; aga.serwin@gmail.com

**Keywords:** antioxidants, bioactive compounds, precocious puberty, overweight, obesity, polyphenols, flavanols, isoflavones

## Abstract

**Background/Objectives**: Precocious puberty in girls currently appears to be one of the main problems in pediatric endocrine gynecology. Early onset of menstruation (EOM) means that the age at which the first menstruation occurs is lower than the average/median for the population, which ranges from 12 to 13 years and depends primarily on ethnic origin. Depending on age and severity of symptoms, these disorders negatively affect girls’ quality of life in many areas, including school life, family relationships, and everyday life. **Methods**: This article provides a scoping review summarizing scientific evidence from human studies on the association between substances derived from green tea (flavanols) and soy (isoflavones) and precocious puberty in girls. **Results**: Despite the relatively small number of girls enrolled in the studies, available scientific evidence from randomized controlled trials (RCTs) suggests that polyphenols from decaffeinated green tea (DGTP) may contribute to lowering the age of first menstruation in girls living with obesity. The effect of soy isoflavones or soy in the context of premature menstruation in girls is unclear. Most studies report that it may have no effect on the age of first menstruation, while individual studies suggest that very early exposure to soy (< 4 months of age) may result in earlier puberty, and others suggest that higher consumption of soy isoflavones delays this process. **Conclusions**: Further well-designed intervention studies in humans are needed to better understand the endocrine and metabolic relationships regarding the role and importance of specific polyphenols in the pathogenic mechanisms of the development and treatment of precocious puberty in girls.

## 1. Introduction

In recent decades, there has been a global and significant decrease in the age of onset of the first signs of puberty or the first menstruation in girls [1,2,3,4,5,6]. It is known that this may have adverse implications in terms of public health and increased risk of metabolic syndrome, reproductive system cancers, psychological problems, and short stature in adulthood [7]. At the same time, the mechanisms influencing such significant hormonal fluctuations and precocious puberty, which manifests itself, among other things, in the acceleration of secondary sexual characteristics or the very early onset of the first menstruation, remain unclear [1,2,6]. The causes of the acceleration of puberty are believed to include endocrine-disrupting chemicals (EDCs), environmental pollution, fetal life and early feeding, as well as improvements in living conditions, often associated with excessive body weight [2,6]. Strong evidence indicates that excess body weight plays a major role in triggering premature puberty [6,8,9,10,11,12,13]. Based on cross-sectional studies, precocious puberty in girls has been defined as the onset of secondary sexual characteristics before the age of eight, resulting from premature activation of the hypothalamic–pituitary–gonadal (HPG) axis or other hormonal mechanisms [2], although some epidemiological studies indicate an age between six and seven, depending on ethnic origin [2]. However, these reports have not changed the clinical definition [2]. Therefore, the food components of individual diets can influence precocious puberty in girls through many mechanisms [14,15,16,17]. These effects may be due to their impact on body weight change, appetite control, or oxidative stress markers [1,14,15,16,17]. Due to their high diversity and wide range of associated effects, polyphenols are one of the food groups that may be an important dietary factor in premature puberty in girls [1,14,15,16,17,18,19]. Lignans, which are the primary phytoestrogens in a typical Western diet, are associated with a later age of menarche in overweight girls [18,19]. Similarly, higher intake of flavonols, which represent flavonoids, has been correlated with a later age at menarche, but no association with body mass index (BMI) has been reported [18,19]. Other frequently studied flavonoids include flavanols found in green tea (*Camellia sinensis* (L.) Kuntze) and soy isoflavones, which can be found most commonly in soybeans (*Glycine max* (L.) Merr.) and legumes, but also in many fruits, vegetables, and seeds [16,17,20,21,22,23,24]. Soy isoflavones have been controversial for years due to their structural similarity to estradiol, and their ability to bind estrogen receptors, particularly ERβ [25,26]. Theoretical concerns have been raised about the potential impact on endocrine disruption during critical periods of development, particularly during infancy and childhood [26]. However, clinical and epidemiological studies conducted on humans have yielded inconsistent results, and most longitudinal and interventional studies have not shown any significant hormonal or developmental effects associated with feeding infants soy-based formulas [15,25,26,27]. Furthermore, systematic reviews and meta-analyses have not found consistent evidence to support a causal relationship between dietary isoflavone exposure and changes in puberty [15,26]. It is also known that phytoestrogens have a lower affinity for human estrogen receptors, which means that they have a weaker estrogen-like effect [25]. Despite the relatively small number of clinical studies in this field, there is preliminary evidence from human studies that the inclusion of nutritional interventions involving flavonoids and soy isoflavones may be beneficial in the context of premature puberty in girls, especially those with obesity.

The main objectives of this scoping review were: (a) To review the existing literature on the role of flavanols and isoflavones in precocious puberty in girls, and (b) to draw conclusions regarding the efficacy of EGCG and soy isoflavones and the possible doses with proven preventive or therapeutic effects in the context of early onset of menstruation (EOM).

## 2. Materials and Methods

### 2.1. Search Strategy

We conducted a comprehensive computer search of the PubMed/MEDLINE, CINAH, Scopus, Cochrane Library, and Web of Science electronic databases. The search included English-language randomized clinical trials and observational studies involving humans (cohort studies, case-control studies, and cross-sectional studies) conducted around the world. The search was restricted to peer-reviewed articles published between 1 January 2000 and 1 December 2025 to ensure that the data and research results discussed were accurate and up to date. The following terms related to flavonols or isoflavonols were combined with the terms “early menarche onset” OR “EMO” OR “precocious puberty” to obtain the corresponding peer-reviewed articles: “flavonols” OR “flavanols” OR “EGCG” OR “epigallocatechin-3-gallate” OR “green tea” OR “catechins” OR “isoflavones” OR “soy isoflavones” OR “soy” OR “genistein” OR “daidzein” OR “equol” OR “glycitin’. The search was supplemented by a review of the reference lists and citations of all articles found, as well as additional searches for key authors. The literature search and study selection were conducted independently by two researchers (I.M., and A.S.). Disagreements were resolved through discussion.

#### 2.1.1. Inclusion Criteria

Studies that met the following criteria were eligible for inclusion: (1) Participants were girls; (2) studies presented original results from experimental studies in humans or observational studies (cohort studies, case-control studies, and cross-sectional studies); (3) studies provided clear descriptions of EGCG or soy isoflavones exposure (type and quantity of drink, food or supplements); (4) outcome measures included and described development of pubertal maturity not only during infancy and early childhood (e.g., breast development including Tanner staging, menarche, pubarche or serologically confirmed early puberty or symptoms suggesting early sexual development), or the study included girls diagnosed with central precocious puberty (CPP); and (5) the full text of the article was published in a peer-reviewed journal in the English language.

#### 2.1.2. Exclusion Criteria

**Figure 1:**
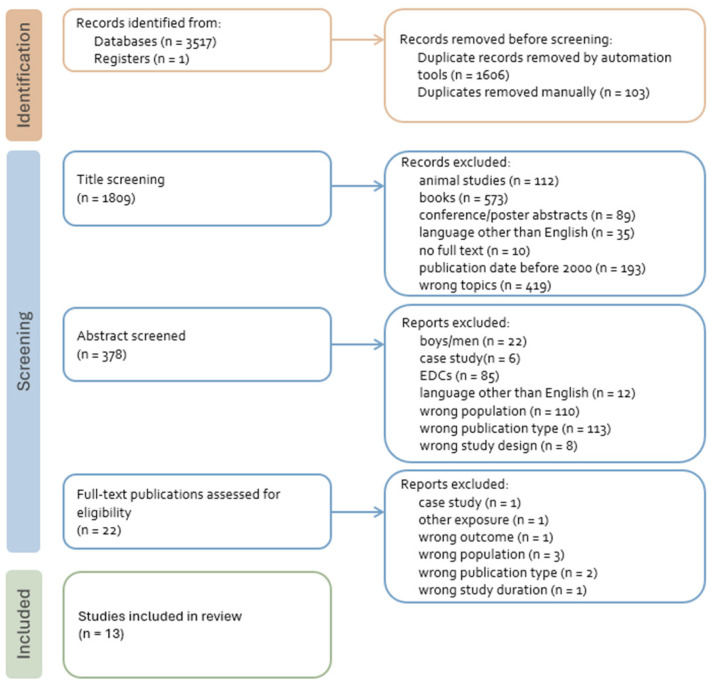
Flowchart of study screening and selection process.

The exclusion criteria were as follows: (1) Participants were boys; (2) articles were: case studies, commentaries, letters to the editor, narrative reviews, review of reviews, in vitro studies, animal studies or books, recommendation and guidance documents; (3) articles presented duplicate data (e.g., same sample as in another article already included in the review); (4) cohorts did not have enough follow-up time; (5) the articles were in a language other than English or were published in other than a peer-reviewed journal; and (6) the data concerned exposure during prenatal life. Studies included in the review are presented in the flowchart of the review and selection process shown in Figure 1.

### 2.2. Data Extraction

Each included study was independently read by two researchers (I.M. and A.S.) to extract the following dimensions: (1) Descriptive information, including authors, year of publication, and type of study; (2) information about participants, including sample size, age, anthropometric parameters (if known), and sample description; (3) information regarding the flavonoids used (EGCG or soy isoflavones), their type (food or supplement) and quantity, as well as placebo control in the case of RCTs; (4) selected information on the development of sexual maturity, including breast development (Tanner age), age at first menstruation, appearance of pubic hair, serological or clinical indicators or early sexual maturation, and ovarian size; (5) type of improvement achieved (delay in puberty or reduced severity of sexual development characteristics) or change in BMI, total weight, or anthropometric parameters as the main outcome measure. Significant information about each study, including authors, years of publication, study samples, methods, and results, is presented in the tables. Formal risk of bias assessment was not performed due to the scoping nature of the review, consistent with JBI and PRISMA-ScR guidance.

## 3. Results

### 3.1. Selected Flavonoids and Precocious Puberty in Girls

**Figure 2:**
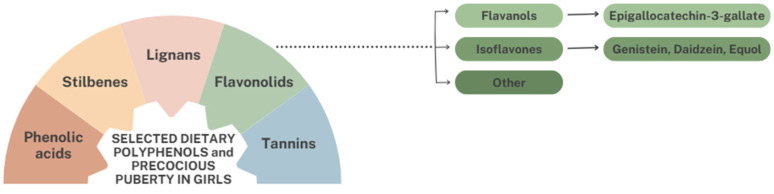
Flavonoids potentially important for precocious puberty in girls.

Polyphenols are organic chemical compounds that are typically found in small amounts in a wide variety of plants [28,29]. They belong to the group of phytochemicals, substances considered to be biologically active in the body [28]. Polyphenolic phytochemicals are a huge group of compounds that can be divided into different classes, the largest of which are flavonoids, comprising a group of over 4000 plant metabolites [28,30]. Flavonoids that are important in the context of premature puberty in girls include flavanols and isoflavones, as illustrated in the figure below (Figure 2).

#### 3.1.1. Flavanols—Polyphenols Contained in Green Tea and Precocious Puberty

Green tea is extremely rich in catechins (flavan-3-ols), which are flavanols, a subclass of polyphenolic flavonoids [20,31]. 100 g of green tea contains up to 13.6 g of catechins, about three times more than black tea [32]. The main catechin, in both quantity and effectiveness, is epigallocatechin 3-gallate (EGCG) [20,31,33]. Other bioactive compounds include flavones and flavonols, primarily known for their anti-cancer properties, such as inhibiting proliferation and angiogenesis in cancer cells [32,33,34]. Due to its structure, EGCG is classified as an antioxidant and, in this respect, is most often studied and used in the context of improving health [33,35,36,37,38,39]. However, the spectrum of its action remains much broader and more complex [20].

The beneficial effect of EGCG on precocious puberty in girls may result from its effect on reducing body weight and markers of inflammation associated with excess body weight [16]. The link between overweight or obesity and low-grade inflammation (LGI) has been proven in numerous studies in both adults and children [40,41,42,43,44]. For this reason, green tea and the flavanols it contains appear to be a promising approach to delaying the age of menarche in girls [16,17]. However, the benefits of weight reduction in children and adolescents suffering from excess body weight appear to extend well beyond the field of endocrine gynecology [42,43,44].

Nowadays, increasing evidence indicates that obesity is an important factor associated with earlier pubertal onset and an increased risk of precocious puberty in girls. Increased BMI and adiposity have been consistently linked to earlier development of secondary sexual characteristics, including breast development and earlier menarche [8,9]. Excess adipose tissue contributes to hormonal and metabolic changes, including increased production of leptin and peripheral conversion of androgens to estrogens, which may accelerate activation of the HPG axis [40,42,44]. Epidemiological studies have demonstrated that girls with higher BMI are more likely to experience earlier pubertal onset compared with healthier-weight peers [8,9,10]. These findings suggest that increased adiposity may act as a permissive or facilitating factor in the initiation of puberty by signaling sufficient energy availability for reproductive maturation [8,9,10]. Higher body fat levels have been associated with the earlier onset of breast development, supporting the hypothesis that obesity contributes to earlier activation of pubertal processes [8,9,10]. Overall, these findings indicate that obesity is not only associated with metabolic disturbances but also plays a significant role in the timing of pubertal development. Increased adiposity may influence endocrine and metabolic pathways involved in pubertal regulation, thereby increasing the risk of earlier pubertal onset and precocious puberty in girls [8,9,10].

**Figure 3:**
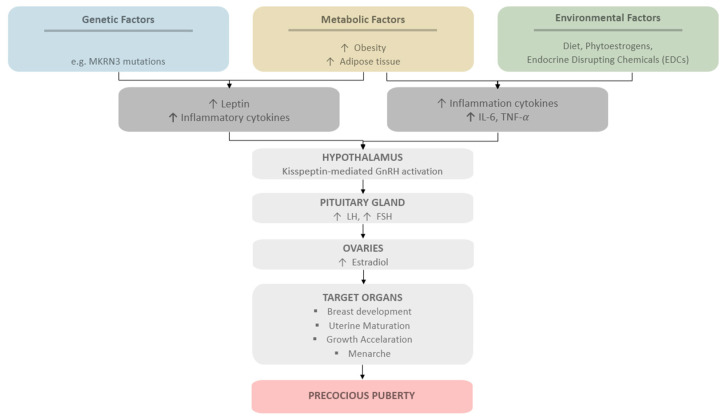
Selected pathways leading to precocious puberty in girls; ↑ indicates an increase.

Simultaneously, chronic low-grade inflammation associated with obesity has been identified as an important factor influencing the onset of puberty. Adipose tissue is not only an energy storage organ, but also an active endocrine organ that secretes pro-inflammatory adipokines and cytokines, including interleukin-6 (IL-6), tumor necrosis factor-α (TNF-α), and leptin [40,41,42]. In overweight children, increased fat mass is associated with elevated levels of inflammatory markers in the blood, indicating a state of chronic, low-grade systemic inflammation [41,42,43,44]. Leptin, predominantly produced by adipocytes, plays a key role in linking energy balance with reproductive function [40,42]. Increased leptin levels in obese individuals reflect energy sufficiency and act as a permissive signal for activation of the HPG axis [42]. This activation leads to increased secretion of luteinizing hormone (LH) and follicle-stimulating hormone (FSH), which in turn stimulates the production of estradiol by the ovaries and promotes the development of secondary sexual characteristics. Elevated leptin concentrations have been associated with earlier pubertal onset and may stimulate hypothalamic pathways involved in gonadotropin-releasing hormone (GnRH) regulation, thereby promoting activation of the reproductive axis [8,9,42]. In particular, leptin and other metabolic signals may influence hypothalamic kisspeptin neurons (KISS1), which act as key upstream regulators of GnRH secretion and play a central role in the initiation of puberty [45,46,47]. Furthermore, obesity-related inflammation and adipokine dysregulation may influence hypothalamic function and neuroendocrine signaling pathways involved in pubertal regulation. Chronic low-grade inflammation and increased adiposity have been associated with earlier pubertal development and menarche, suggesting that metabolic and inflammatory factors contribute to premature activation of the HPG axis in girls [10,12,42]. We have presented a diagram summarizing the factors that can accelerate the maturation process in the graphic below (Figure 3).

Xie et al. proved that polyphenols from decaffeinated green tea (DGTP) at a dose of 400 mg/day can statistically significantly reduce fat mass (FM), fat-free mass (FFM), percentage body fat (PBF), and reduce the volume of both ovaries [16]. The basal metabolic rate also increased in the study group. Probably due to dietary and exercise instructions from qualified dietitians, both groups showed improvements in body mass index (BMI), waist circumference (WC), waist-to-hip ratio (WHR), waist-to-height ratio (WHtR), and serum uric acid (UA) concentration [16]. A significant improvement has therefore been demonstrated in terms of obesity reduction and metabolic health (uric acid), but the effect on the timing of puberty is not entirely clear. However, in the study group, ovarian size decreased and sex hormone concentrations increased over the 12-week intervention. Although the increase in hormones was statistically significant, the absolute levels remained low—only slightly above the typical concentrations observed during puberty in girls. This suggests that the girls with obesity included in the study had not yet entered this phase of sexual development, indicating that puberty was not accelerated and may have been minimally delayed, despite obesity [16].

In another study, which combined clinical trials with animal models, researchers continued to administer DGTP at a reduced dose of 200 mg/day in the clinical part [17]. In girls from the study group, an increase in neurokinin B (NKB) and estradiol (E_2_) levels and a decrease in left ovary volume were observed [17]. In addition, serum NKB levels were statistically significantly lower than in the placebo group by 0.599 ng/mL [17]. The observed hormonal and ovarian changes may help explain a potential delay in puberty among girls with obesity [17].

One of the main limitations of both studies is that they examine green tea polyphenols as a whole, rather than isolating individual active substances. Although it is known that EGCG is the most abundant catechin in green tea and has a proven effect on reducing body weight, body fat, and cholesterol in adults, it cannot be conclusively stated that this is due to EGCG alone, rather than other polyphenols derived from it [16,17,48,49]. It is therefore possible that the effect of using EGCG alone would not be as significant as when using a larger number of polyphenols. One Japanese study on green tea catechins showed improvement in obesity and a reduction in leptin levels in children living with obesity [50].

To present the current state of knowledge on the impact of selected flavanols on the occurrence of premature puberty in girls, the results of the included clinical studies are summarized below (Table 1).

#### 3.1.2. Soy and Soy Isoflavones and Precocious Puberty

Soy isoflavones, along with lignans and coumestans, are classified as one of the main groups of phytoestrogens [51]. Phytoestrogens, or natural polyphenols, are active substances of plant origin that exhibit affinity for estrogen receptors due to their structural similarity to 17-β-estradiol (E2) [51,52,53,54]. The main sources of soy isoflavones in food are considered to be soybeans and soy products, along with various types of beans, broad beans, broccoli, asparagus, nuts, and seeds [21,22,23]. Due to their similar structure, phytoestrogens bind to estrogen receptors and can exhibit both estrogenic and antiestrogenic effects [55]. Their weaker effect compared to E2 is often emphasized [54,55]. The literature also typically emphasizes their diverse and selective actions depending on the tissues and the receptor located on them: the estrogen receptor alpha (ERα) or estrogen receptor beta (ERβ) [54,55,56,57]. For this reason, they are often referred to as selective estrogen receptor modulators (SERM-like) [56,57]. Phytoestrogens are credited with a number of health benefits, including reducing the risk of diet-related diseases, such as cardiovascular disease, type 2 diabetes, and obesity, as well as cancers of breast, prostate and colon [53,58,59,60,61,62,63,64,65]. Due to their beneficial effect on reducing menopausal symptoms, including osteoporosis and hot flushes, phytoestrogens are also indicated as an alternative or adjunct to hormone replacement therapy for menopause [53,66]. In addition to their antioxidant effects, soy isoflavones may help with weight loss by reducing inflammation, which is often elevated in individuals with excess weight or obesity [67,68]. Soy isoflavones may also influence weight loss and improve the lipid profile in adults. They may also normalize the expression of many liver genes responsible for lipid metabolism, thereby improving metabolic parameters [69,70].

The link between soy and early puberty appears to be multifaceted. On the one hand, there are fierce debates about whether exposure to soy during infancy or early childhood can cause hormonal disorders in children. On the other hand, soy may play a role in modulating inflammation, influencing metabolic parameters, and affecting body composition [71,72,73,74,75,76,77,78,79,80].

Clinical trials involving pre-pubertal girls or large observational studies have yielded inconsistent findings [71,72,73,74,75,76,77,78,79,80]. Strom et al. (2001) and Adgent et al. (2012) conducted cohort studies to examine the relationship between exposure to soy-based formula in early childhood and the age of first menstruation onset or the acceleration of puberty symptoms [71,72]. In their retrospective study, Strom et al. did not observe that feeding soy-based formula (*n* = 128) in early childhood had a statistically significant effect on the age of first menstruation or accelerated breast development compared to women fed with cow’s milk-based formulas (*n* = 268) [71]. In comparison, the results of a study by Adgent et al. indicated that girls fed soy-based milk substitutes introduced before four months of age (early soy) had an approximately 25% higher risk of early onset of menarche compared to other groups: primarily breast, early formula (various types of non-soy-based formulas), and late soy [72]. A slight reduction in risk was associated with breastfeeding [72]. It is worth emphasizing that this study found no differences in childhood BMI z-scores or the prevalence of overweight between the early soy and early formula groups. This means that the relationship between the earlier onset of menstruation in the group of girls who were fed soy-based formula early on and the other groups was not influenced by BMI; i.e., it was not due to excess weight [72].

In a study of 45 girls by Sinai et al. (2019), anthropometric parameters (height, BMI z-score) did not differ significantly between participants fed soy-based formula (*n* = 12) and those fed cow’s milk-based formula (*n* = 33) [73]. One girl from the soy-consuming group and eight girls from the control group showed early signs of puberty, but after taking into account BMI and family data, no correlation was found between puberty and the type of milk substitute used in infants [73]. In this prospective study, no association with puberty was found, nor were there any differences between groups in terms of current daily soy intake, energy, macronutrients, or micronutrients obtained from patients’ food diaries at the time of assessment at 7.8–10.5 years of age [73].

Most concerns about soy focus on its impact on the early onset of menarche. Cheng et al. (2010) demonstrated in an observational prospective cohort study that girls who consumed higher amounts of isoflavones before puberty experienced later onset of puberty [74]. This applied to both the later age at which girls reached Tanner stage 2 breast development and peak height velocity (PHV) [74]. Higher isoflavone intake was also associated with later age at take-off (ATO). However, the conclusions drawn by the authors of the study emphasize that no statistically significant difference was found between the age of menarche and the level of isoflavone consumption, with the trend indicating a slightly later onset with higher soy consumption [74].

Segovia-Siapco et al. (2014) also conducted a cross-sectional study to examine whether soy consumption in the diet is associated with the age at onset of menarche (AOM) in a population of girls exposed to significant amounts of soy in their diet (Seventh-day Adventists) [5]. The results of the study indicate no association between soy consumption among adolescents and AOM. Also in this study, as in the studies by Adgent et al. and Sinai et al., BMI and BMI with z-score were taken into account when selecting the control sample or in terms of the groups participating in the study, which suggests the absence of a confounding variable in the form of the influence of excessive body weight alone on the study results in any of the groups [5,72,73]. However, the authors suggest that higher soy consumption and the growing popularity of certain soy products may not be related to the trend of premature sexual maturation observed for many years, mainly the lower age of onset of menarche in girls [5].

Kim et al. (2014) conducted a case-control study with age-matched controls involving 199 girls, of whom 108 had idiopathic central precocious puberty [20]. This study examined whether serum isoflavone concentration is associated with central precocious puberty (CPP) in a population of Korean girls [20]. In girls with CPP, concentrations of daidzein, genistein, and total isoflavones were elevated compared with those in the control group [20]. The incidence of CPP was also statistically higher among girls whose serum isoflavone concentration was ≥30 nmol/L, compared with those below this threshold [20]. However, the authors do not make any hasty conclusions. They suggest that serum isoflavone concentrations may be associated with the risk of precocious puberty in girls in the study population, while emphasizing the need for further long-term observational studies and randomized controlled trials [20].

A retrospective, case-control study conducted by Felício et al. aimed to investigate the association of CPP not only with soy but also with exclusive breastfeeding (EBF) [75]. EBF was less common among girls in the study group, and this proved to be a protective factor in the context of CPP. At the same time, soy consumption was significantly higher in the group of girls affected by premature puberty and correlated with its prevalence [75]. This Brazilian observational study showed that soy intake was associated with CPP and that EBF was a protective factor. At the same time, the researchers emphasized the need for more methodologically sound studies, including observational prospective studies or RCTs [75].

The studies by Andres et al. and Gilchrist et al., which were conducted as part of the prospective cohort of the Beginnings study, reported that at both four months and five years of age, there were no statistically significant differences in anthropometric measurements or body composition between girls fed using different methods, including breast milk (BF), cow milk-based formulas (MFs), or soy-based formulas (SFs) [76,77]. At both time points, no differences in breast or uterine size were found among the girls studied [76,77]. Female infants fed MF had a larger mean ovarian volume (*p* < 0.05) and a greater number of ovarian cysts per ovary (*p* < 0.01) than those who were breastfed [77]. Moreover, at the age of five, no significant changes were observed in girls in terms of ovary size, number of ovaries with cysts, number and size of ovarian cysts, and uterus shape [76]. Although there are indications that MF feeding may accelerate ovarian development, no effect of SF on the reproductive organs studied in girls has been reported [76,77]. However, the study is ongoing, and the authors continue to observe the girls and possible links in terms of accelerated or delayed age of onset of menarche among participants from different childhood nutrition groups [76,77,78].

The most recent study included in the review concerned the relationship between soy and dietary fiber intake and their impact on the timing of sexual maturation [79]. Among the girls included in the study, variables such as anthropometric data, age at Tanner stage 2 for breast development (B2), and age at the initiation of gonadal growth (G2) or age at menarche (M) were assessed, which may indicate accelerated sexual maturation [79]. Urine samples were also tested for equol, an active metabolite of soy. The results showed that among girls, higher soy intake was associated with a statistically significant later onset of puberty, regardless of prepubertal body fat and fiber intake [79]. Hazard ratio (HR)-B2: 0.88 (95% CI, 0.80–0.96), *p* = 0.02; HR-M, 0.87 (0.77–0.94), *p* = 0.01; HR-G2, 0.91 (0.82–0.98), *p* = 0.013; HR-VB, 0.90 (0.82–0.9), *p* = 0.02). It was also emphasized that these relationships were more pronounced among children with high levels of equol in their urine (*p*_for-interaction_ ≤ 0.04) or high intake of cereal fiber (*p*_for-interaction_ ≤ 0.06). Dietary fiber intake or its subtype was not prospectively associated with the onset of puberty after adjusting for soy intake in the diet (*p* ≥ 0.06) [79]. Due to the study’s nature, it is not possible to talk about a cause-and-effect relationship here, but the authors of the study emphasize that higher soy consumption in childhood is prospectively associated with later onset of puberty in Chinese girls. This association is particularly pronounced in individuals with higher levels of equol in their urine [79]. It is also worth noting that this observation is independent of pre-pubertal body fat content [79].

At this point, it is worth noting that equol is the most metabolically active metabolite of daidzein, which exhibits higher biological activity than both major soy isoflavones, daidzein and genistein [80]. According to research, only 25–50% of the population is capable of converting daidzein into equol with the help of intestinal bacteria [81,82,83]. Most people can only produce a hormonally inactive compound: O-desmethylangolensine [81,82,83]. Studies report that an increase in equol production can be observed in the Asian population, as well as among vegetarians [82,84]. These data may suggest the influence of the habitual diet on the potential increase in the ability of the intestinal microbiota to produce equol from daidzein [82]. Bacteria capable of producing equol include numerous bacteria from the *Coriobacteriaceae* family, but also some strains from the *Bifidobacterium*, *Lactobacillus*, and *Lactococcus* genera [85]. Both of the aforementioned phytoestrogens, daidzein and equol, have the ability to bind to ERα and ERβ [86]. The affinity of the daidzein metabolite for estrogen receptors is also higher than that of daidzein itself [86]. ERα and ERβ receptors are unevenly distributed in different tissues, which means that the effect of equol, having a higher affinity for the latter of these receptors, also varies [86]. ERβ is mainly found in the ovaries, kidneys, colon, central nervous system (CNS), and cardiovascular system, and it is there that the greatest effects of its action would be expected [87]. Another variable influencing the action of isoflavones or their metabolites is the aforementioned fact of greater affinity for receptors by endogenous estrogens [25,85]. In the context of premature puberty and the excessive body weight that often accompanies it, the ability of daidzein and equol to neutralize reactive oxygen species and prevent oxidative stress, mainly through the activation of antioxidant enzymes, is particularly noteworthy [88].

Only one study meeting the inclusion criteria for the review was a randomized controlled trial [89]. This was a study by Duitam et al., which included a group of only 27 girls and lasted as long as 12 months [89]. Girls aged 7–9 years from the study group were given fruit juice with 45 g of a commercial soy protein-based supplement (SPS) added every day, while girls from the control group were given the same juice without the SPS [89]. Statistically significant differences after the intervention between the study and control groups of girls were observed in the context of height and the following indices: BMI/age, weight/age, and height/age [89]. No changes in Tanner stage maturity were observed in any of the girls during the study period—all remained at stage 1 [89]. The conclusions emphasized that long-term dietary supplementation with 45 g of soy-based supplement did not affect sexual maturation or the onset of puberty in prepubertal girls [89]. However, at the same time, this supplementation may influence an increase in height, BMI/age, height/age, and weight/age in girls, which was associated with changes in lean body mass [89].

To present the current state of knowledge on the relationship between soy or isoflavones and the occurrence of premature puberty in girls, the following table was constructed, containing the included clinical and observational studies (Table 2).

## 4. Limitations and Future Research

Considering the increasing prevalence of precocious puberty and the global trend toward earlier onset of menarche among girls, as well as the limited information available on the impact of food and supplements on this condition, this paper provides a comprehensive summary of the available literature on the role of flavanols and isoflavones in the context of this disorder. Decaffeinated green tea polyphenols and soy appear to act through other mechanisms on premature puberty in girls. According to RCTs, DGTP may delay the age of menarche by helping to control body weight in overweight girls, although these studies have been conducted on a relatively small population in Asian countries [16,17]. Soy isoflavones, on the other hand, do not appear to accelerate this phenomenon and may even delay it in some populations, although the evidence so far comes mainly from observational studies or small clinical trials [5,71,73,74,79,89].

This article has numerous strengths. First, our article summarizes the current state of knowledge on the use of selected polyphenols in the context of precocious puberty. The paper may also indicate the direction of further research into the use of selected groups of polyphenols in the context of weight reduction and improvement of metabolic parameters in young girls whose premature puberty is associated with overweight or even obesity. Decaffeinated EGCG may contribute to delaying the onset of menarche in this population [16,17]. There is limited data on the effect of soy isoflavone consumption on delaying the onset of menarche [74,79]. According to the literature, this can be achieved by influencing various mechanisms, including weight reduction, improvement in anthropometric parameters (↓ WC, ↓ WHR, ↓ WHtR, ↓ fat mass) or metabolic parameters (↓ uric acid serum level), and reduction in ovarian volume in studies on DGTP [16,17].

In contrast to aforementioned common concerns about the impact of soy on hormonal disorders in children, the majority of studies assessing increased soy exposure do not demonstrate a causal association with earlier pubertal onset or reduced age at menarche in girls [5,27,71,73,79,89]. It is worth noting that the studies that found no effect of soy isoflavones on early menstruation were randomized prospective observational studies, retrospective observational studies, and case-control studies embedded in a prospective cohort study [5,71,73,79,89]. Meanwhile, studies suggesting their potential impact on accelerating menstruation include a case-control study, a cohort study, and a retrospective case-control study [20,72,75]. One study observed a modest reduction in age at menarche associated with early soy exposure (<4 months of age), whereas no such association was identified with habitual dietary intake of soy isoflavones [72]. The study also showed that breastfeeding reduced the risk of early menstruation [72]. The protective effect of exclusive breastfeeding until 6 months of age on the risk of CPP was also confirmed by a team led by Felício [75], although the same study showed a link between soy intake and CPP [75].

The effect of soy on delaying age at take-off and peak height velocity may have a similar significance for premature menstruation as DGTP, exerting an indirect effect, i.e., by appropriately reducing the growth rate (isoflavones) and body weight (soy isoflavones) [16,17,74]. Conversely, one study reports that 12 months of soy supplement consumption does not affect age at menarche (AOM) but is associated with higher BMI/age, weight/age, height-for-age, and weight-for-age indices [89]. Therefore, these studies yield conflicting conclusions, and it is currently difficult to determine whether adding soy to the diet can have a beneficial or adverse effect on body weight, height, or BMI [16,17,74,79,89]. This is quite an important issue, given that excessive body weight correlates positively with precocious puberty [6,8,9,10,11,12,13].

In future studies, it is also worth paying particular attention to the degree of soy processing in the studies conducted, as higher consumption may be associated with a more processed diet, which in itself may lead to weight gain due to a higher supply of kilocalories [18,19]. Only one of the studies included in the review differentiated between sources of soy: soy beverages, traditional soy/tofu, and meat alternatives [5]. The implementation of this classification made it possible to observe the different effects of soy products with varying degrees of processing [5].

Soy isoflavones are known to support the treatment of various metabolic diseases in adults, including polycystic ovary syndrome [69,70,90,91,92,93]. They have also been linked to weight loss by reducing inflammation, including LGI, which often accompanies excess weight or obesity [67,68]. Given that precocious puberty is most commonly associated with excess weight and potential metabolic disorders, as well as LGI, it would be reasonable to design studies involving soy isoflavones that take into account their impact on anthropometric and metabolic parameters in a group of girls affected by precocious puberty [16,17,90,91,92,93].

Furthermore, our review has the advantage of including clinical trials involving girls or cohort studies involving populations of girls around puberty, and does not include animal studies, in which the metabolism of EGCG or soy may differ significantly at doses considered effective for both supplements and dietary intake. However, it is worth noting that this kind of review does not take into account the quality of the studies included in it as thoroughly as a systematic review would. This may lead to conclusions that could change or lose their significance after a qualitative assessment of the studies included.

The review includes several high-quality studies, including prospective cohorts and randomized clinical trials involving girls [16,17,73,74,79,89]. However, the populations studied are quite small and often do not include ethnic diversity, so it is unclear whether the findings can be extrapolated to the general population of prepubescent girls. The review also included retrospective observational studies and cross-sectional studies involving larger numbers of participants, but these provided less reliable data and lacked reliable cause-and-effect relationships due to methodological limitations [71,75]. It is also worth holding off on making hasty conclusions about soy, as studies suggesting delayed puberty in populations of girls consuming large amounts of soy refer to populations with a high habitual intake [5,79]. The methodology of previous studies involving isoflavones is highly variable, and there is a lack of well-designed clinical control studies supported by specific biomarkers, such as isoflavone concentrations in urine or serum. Differences in exposure assessment methods may also contribute to inconsistent results. Studies using dietary questionnaires may be less precise than studies using objective biomarkers such as urinary or serum isoflavone concentrations. In addition, variability in timing and duration of exposure, particularly during sensitive periods of development such as early childhood, may influence the observed effects. Observational or cross-sectional studies limit causal inferences and may be subject to memory bias and misclassification of exposure. In contrast, prospective cohort studies with long-term follow-up typically provide more reliable evidence because they allow for the establishment of temporal relationships between exposure and puberty outcomes.

Biological heterogeneity, including differences in isoflavone metabolism and equol production status, obesity, and baseline hormonal environment, may further modify individual susceptibility to the endocrine effects of phytoestrogens. Furthermore, inadequate adjustment for confounding factors such as BMI, total energy intake, and environmental exposure may partly explain the inconsistent results to date. It would therefore be necessary to consider and confirm in well-designed studies whether similar results would be found in populations where soy consumption remains low and equol metabolism is probably limited. The authors suggest that research in the context of soy should be divided into prospective cohort studies that can confirm or refute the safety of using milk substitutes and soy products in early childhood and RCTs. However, it is also important to address confounding factors and select uniform and easily reproducible protocols and outcome measures, preferably in the form of biological markers.

On the other hand, based on the promising results achieved in the context of clinical trials using DGTP in this group of subjects, it would be worth considering designing analogous RCTs with specific doses of soy isoflavones. We believe that studies designed in this way would be reproducible and could provide interesting data. We are referring to studies involving overweight girls aged 6–10 years with a specifically defined dose of soy isoflavones and simultaneous measurement of markers of inflammation, IGF-1, leptin, and anthropometric parameters and medical classifications assessing the physical stages of puberty. This is related to the fact that in adults who are overweight or obese, soy isoflavones have been shown to have beneficial effects in terms of both inflammation markers and body weight, as well as the risk of diet-related diseases.

It is also worth noting that studies involving the use of milk substitutes are often limited to a single country. This is a potential disadvantage, as there are no uniform guidelines, apart from the general information provided in the *Codex Alimentarius*, on the regulation of the composition of milk substitutes on a global scale [94]. In Europe, these guidelines are regulated by Regulation (EU) 2016/127, in the US by the Federal Drug Administration guidelines, and in Australia and New Zealand by the recently modified Australian-only Infant Formula Product Standard [95,96,97]. Despite their similar formulation, these guidelines do not guarantee uniformity in the composition of soy-based milk substitutes used in the nutrition of infants and young children. Therefore, it seems that in the absence of uniform guidelines, it is impossible to compare studies using soy-based milk replacers.

A potential limitation of some of the studies included in the review is that research on DGTP, suggesting a promising effect on delaying puberty in girls, was conducted exclusively on Asian girls [16,17]. However, similarly, the only study that suggested soy’s effect on accelerating the age of menarche was also a study conducted exclusively in Asia [20]. Ethnic and geographic differences in phytoestrogen intake may be particularly relevant for drawing conclusions about the impact of soy on puberty, as populations living in East Asia or members of the Seventh-day Adventists typically consume significantly higher amounts of soy isoflavones compared to Western populations. These dietary differences may contribute to variability in endocrine exposure and timing of sexual maturation and should be considered when interpreting observational studies.

At the same time, a potential drawback of some of the studies included in the review appears to be the lack of a uniform diagnosis and the diversity of endpoints. In some studies, girls in the study groups are diagnosed with CPP [20,75]. In others, there is no clear diagnosis, but symptoms of precocious puberty are observed, and the endpoints are either the time of the first menstruation, changes in the Tanner scale, or anthropometric parameters, including body weight, height, BMI, or PHV [71,72,74,75]. Research on DCGP focuses on improving anthropometric and metabolic parameters (uric acid) [16,17]. In turn, studies on soybeans examine either the concentrations of the main soy isoflavones or their total concentration, or their effect on AOM, PHV, or ATO [5,20,75].

The above studies do not provide a clear answer to the question of their use in preventing premature puberty in girls, although data from DCGP studies appear promising [16,17]. However, further, more detailed research is needed to confirm the usefulness of these polyphenols in preventing premature menstruation in girls and to formulate clear guidelines. Multicenter trials involving centers in different parts of the world would be desirable in order to ensure a relatively large sample size and ethnic diversity of the study group. It is also recommended to use standardized preparations containing EGCG or soy isoflavones to exclude the influence of dosage or accompanying substances. There would also be good reason to investigate the impact of these substances on markers of inflammation, especially in girls who are overweight or diagnosed with obesity.

**Figure 4:**
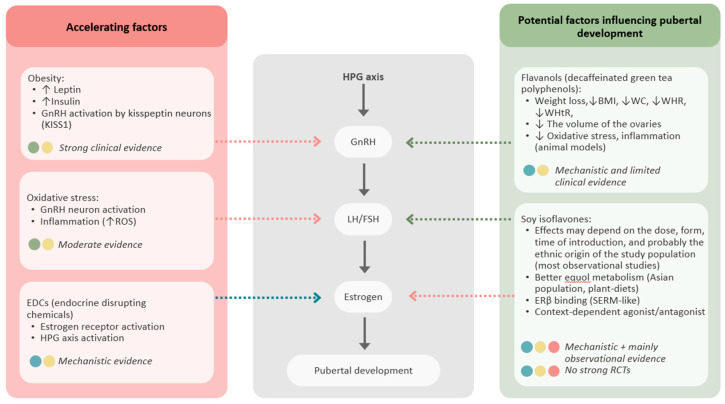
Potential influence of flavanols and soy isoflavones on HPG axis and pubertal timing. BMI—body mass index; ERβ—estrogen receptor beta; FSH—follicle-stimulating hormone; GnRH—gonadotropin-releasing hormone; HPG—hypothalamic–pituitary–gonadal; KISS1—kisspeptin; LH—luteinizing hormone; RCTs—randomized controlled trials; ROS—reactive oxygen species; SERM—selective estrogen receptor modulator; WC—waist circumference; WHR—waist-to-hip ratio; WHtR—waist-to-height ratio, ↑ indicates an increase; ↓ indicates a decrease.

Mechanistic evidence suggests that flavanols and isoflavones may influence premature puberty at various stages of the HPG axis; for example, by affecting estrogen receptor signaling or modulating oxidative stress. However, clinical evidence confirming their causal role in the timing of sexual maturation remains limited. We have provided a graph below summarizing the factors that may accelerate the maturation process and those that may potentially delay it in girls (Figure 4).

## 5. Conclusions

Flavanols and isoflavones found in food, in particular epigallocatechin gallate and soy isoflavones, may be associated with the onset of premature puberty in girls. Among other things, DGTP has the potential to lower BMI, WC, WHR, WHtR, improve body composition, reduce serum NKB levels, and decrease ovarian volume, which may result in delayed puberty in prepubescent girls affected by obesity. Studies to date have been conducted on groups of girls aged 6–10 years. Observing the global trend of excessive weight in children and the clinical definition of premature puberty in girls as <8 years of age, it may be worth considering intervention even in girls aged 4–5 years if they are accompanied by obesity. Effective doses in studies are as low as 200 mg of DGTP per day in the context of reducing breast volume, increasing kisspeptin, and lowering IGF-1 and NKB levels in the study group. In turn, at a dose of 400 mg DGTP per day, benefits such as a decrease in BMI, WC, WHR, WHtR, and a reduction in ovarian volume were observed. Further placebo-controlled clinical trials should therefore be conducted on similar premises, but certainly with a larger number of girls with a more diverse ethnic background, as both RCTs conducted to date have been carried out in Asian countries.

Moreover, the relationship between soy isoflavones and precocious puberty in girls is unclear. Most studies in this area indicate that both soy consumption in early infancy and during puberty or pre-puberty does not accelerate the onset of the first menstruation [5,73,74]. Some studies, although observational, suggest that higher soy consumption may delay this age. The greatest concern is early exposure to soy, i.e., before the age of four months, because Adgent et al. observed that it may be associated with a slight but statistically significant acceleration in the onset of menarche [72]. Nevertheless, it appears that the scenario of introducing milk substitutes in early infancy concerns only a small percentage of children (those with galactosemia, congenital lactase deficiency, religious or ethical considerations, and some children allergic to cow’s milk protein). Public concern is raised more by the consumption of conventional soy-based foods in childhood than by their consumption in the form of plant-based milk substitutes. In the context of soy, it is therefore difficult to determine the dose that could delay the age of first menstruation.

Although precocious puberty is currently a problem affecting an increasing percentage of girls, there is a lack of consistent recommendations regarding nutritional and supplementation strategies, making it difficult for researchers and practitioners to effectively help alleviate symptoms or prevent this phenomenon in young girls around the world.

While the research results presented in this article are promising, their relatively small number and methodological limitations, including the type of study and small sample size, currently prevent the formulation of clear clinical recommendations for physicians or dietitians. It is therefore necessary to conduct further, carefully designed clinical trials involving pre-pubertal girls of different ethnic backgrounds to assess the efficacy and safety of both consumption and possible supplementation of DGTP or soy isoflavones in wider populations.

## Figures and Tables

**Table 1:** Characteristics of included studies evaluating the effect of selected flavanols on precocious puberty in girls.

Authors, Year, Country	Study Design, Duration	Characteristics of the Study Group and Intervention	Aim of the Study Nutrient	Main Results	Conclusions
Xie et al., 2021 [16] China	Randomized, double-blinded, placebo-controlled 12-week trial	Participants: 62 girls with obesity Age: 6–10 years Study group: *n* = 31, received 400 mg/day DGTP, orally Control group *n* = 31, received isodose placebo, orally All participants received the same instruction on diet and exercise from trained dietitians	To evaluate the impact of DGTP on obesity and sexual development in girls with obesity aged 6–10 years and to assess the safety profile of this therapy.	↓ BMI, ↓ WC, ↓ WHR, ↓ WHtR, in both groups, ↓ PBF, Fat mass, FFM in study group ↑ Basal metabolic rate in study group ↑ The number of participants with breast Tanner stage II or above and the uterine volume in both groups ↓ The volume of the ovaries on both sides in study group ↓ Serum UA concentration in study group	Short-term daily DGTP consumption significantly improved BMI and body composition and reduced the risk of hyperuricemia among girls with obesity. By reducing ovarian volume, DGCP may be effective in preventing premature puberty in girls living with obesity.
Gu et al., 2022 [17] China	Randomized, double-blinded, placebo-controlled trial—follow-up 12-week	Participants: 50 girls with obesity Age: 6–10 years Study group: *n* = 20, received 200 mg/day DGTP, orally Control group *n* = 30, received isodose placebo, orally All participants received the same instruction on diet and exercise from trained dietitians	To evaluate the impact of DGTP (50% of the total dose used in the previous study) on obesity and sexual development in girls with obesity aged 6–10 years and to assess the safety profile of this therapy.	↑ Serum NKB level in both groups ↑ E_2_ level in both groups ↓ The volume of the left ovary in study group ↑ Kisspeptin level in placebo group ↓ IGF-1 level in placebo group No statistically significant changes in ghrelin or leptin levels in both groups. The serum NKB level in the EGCG group was statistically significantly lower than in the placebo group by 0.599 ng/mL after the intervention and after adjusting for confounding factors.	Supplementation of decaffeinated EGCG may reduce serum NKB levels, which may result in delayed puberty in young girls affected by obesity. DGCP may also be effective in preventing premature puberty in girls living with obesity by reducing the volume of the ovaries.

BMI—body mass index; DGTP—decaffeinated green tea polyphenols; E_2_—estradiol; FFM—fat-free mass; IGF-1—insulin-like growth factor-1; NKB—neurokinin B; PBF—percentage of body fat; UA—uric acid; WC—waist circumference; WHR—waist-to-hip ratio; WHtR—waist-to-height ratio, ↑ indicates an increase; ↓ indicates a decrease.

**Table 2:** Characteristics of included study trials evaluating the effect of soy or soy isoflavones on precocious puberty in girls.

Authors, Year, Country	Study Design, Duration	Characteristic of the Study Group and Intervention	Aim of the Study	Main Results	Conclusions
Strom et al., 2001 [71] USA	Observational retrospective cohort study	Participants: *n* = 396 women Age: 20–34 Exposed: *n* = 128 soy formula as infant Unexposed: *n* = 268 cow milk formula as infant Men also participated in the cohort, but their results were not included in the article.	To investigate whether there is a link between the consumption of soy-based formula by infants and the health status of young adults, particularly their reproductive health.	No statistically significant difference in the age of first menstruation or breast development was observed between girls fed soy formula as infants and those fed cow milk formula.	The results of this study are reassuring in terms of the safety of soy products for infants and their possible impact on premature puberty in girls. However, it should be emphasized that this is a cohort study, not an intervention study, so the quality of the evidence obtained is weaker.
Cheng et al., 2010 [74] Germany	Observational prospective cohort study	Participants: *n* = 119 girls Age: 8–14 years Girls divided into groups according to tertiles (T) of dietary isoflavone intake at the start of the study: T1: *n* = 39—isoflavone intake 13 μd/d T2: *n* = 40—isoflavone intake 49 μd/d T3: *n* = 40—isoflavone intake 1199 μd/d Boys also participated in the study, but their results were not included in the article (108 boys, aged 10–16 years).	To examine whether the intake of isoflavones and fiber by healthy white children before the growth spurt prior to puberty (age at take-off (ATO)) is associated with the timing of the onset of puberty.	Girls who had the lowest dietary isoflavone intake at the start of the study reached PHV approximately 0.6 years earlier and reached Tanner stage 2 breast development approximately 0.7 years earlier than girls who had the highest isoflavone intake [95% CI, *p* = 0.04). Lower isoflavone intake in girls was associated with earlier ATO. These associations remained after adjusting for BMI and fiber intake at the start of the study (adjusted model). No statistically significant difference in age at menarche was found between girls in the lowest and highest tertiles of isoflavone intake, although the trend indicated a slightly later menarche with higher isoflavone intake.	Girls who consume higher amounts of isoflavones before puberty appear to enter puberty at a later age. Fiber intake in this group of healthy white girls did not affect the timing of puberty.
Kim et al., 2014 [20] Korea	Case-control study with age-matched control	Participants: *n* = 199 girls Age: 6–10 years Study group: *n* = 108 girls with idiopathic central precocious puberty (CPP) Control group: *n* = 91 age-matched healthy girls without any symptoms of precocious puberty.	To investigate the association between serum isoflavone concentration and the risk of CPP in Korean girls.	Serum concentrations of daidzein (*p* = 0.0202), genistein (*p* = 0.0021), and total isoflavones (*p* = 0.0009) were ↑ in children with CPP than in the control group. The prevalence of CPP was statistically significantly (*p* = 0.0008) higher in girls with serum isoflavone levels ≥30 nmol/L than in girls with serum levels <30 nmol/L.	Higher serum soy isoflavone concentrations may be associated with the risk of premature puberty in girls in the Korean population. There is a need for long-term observational studies or randomized controlled trials.
Adgent et al., 2012 [72] UK	Longitudinal cohort study (ALSPAC)	Participants: *n* = 2920 infant girls from the entire ALSPAC cohort had sufficient data on infant feeding and puberty to be included in this analysis. Exposed: *n* = 54 from 2920 white girls from the Avon region (UK), healthy or with minor health issues, fed soy-based formulas (early, <4 month of age) Unexposed: girls from ALSPAC cohort, fed non-soy based infant formula or milk (early formula).	Analysis of the timing of the onset of menarche in relation to infant feeding methods, with particular emphasis on the potential impact of soy isoflavones contained in soy-based infant formula.	The risk of early onset of menarche among girls in the early soy formula feeding group was approximately 1.25 times higher (95% CI, 0.92, 1.71) than among girls in the early formula feeding group. A slight reduction in this risk was associated with breastfeeding (HR: 0.95 (95% CI, 0.84, 1.06)).	The study provided preliminary results suggesting that exposure to soy products in early infancy may contribute to a slight increase in the risk of early menarche compared to girls who were mainly breastfed (12.4 vs. 12.8 years). Late soy feeding throughout the observation period did not affect the age of first menstruation.
Segovia-Siapco et al., 2014 [5] USA	Cross-sectional study	Participants: *n* = 327 girls with complete information on soy intake and AOM. Age: 12–18 years Girls divided into groups according to categories of total soy consumption level: Exposed: (A) >3×/day: *n* = 69; (B) >1×/day–≤3×/day: *n* = 111; (C) >1×/week–≤1×/day: *n* = 85; (D) ≤1×/week: *n* = 62.	To determine whether soy consumption is associated with AOM in a population of girls exposed to significant soy consumption.	In girls who reached sexual maturity before or during the study, the AOM was 12.5 ± 1.4 years. Total soy intake and intake of the three specified types of soy products were not significantly associated with AOM, nor did they affect early or late AOM. Adjustment for demographic and dietary factors did not change the results. BMI and BMI z-score did not differ significantly in individual soy consumption groups.	The results of the study indicate that soy consumption during adolescence is not associated with AOM. The authors suggest that higher consumption and growing popularity of soy products may not be related to the long-term downward trend in the age of first menstruation among girls.
Andres et al., 2015 [76] Gilchrist et al., 2010 [77] USA	A long-term cohort	Participants: *n* = 51 girls Aged: 4 months, 5 years Girls divided into groups depending on feeding method as infants: BF: *n* = 18 MFs: *n* = 18 SFs: *n* = 15 Boys also participated in the study, but their results were not included in the article (50 boys, aged 4 months, 5 years).	Whether there are changes in the development of reproductive organs (assessed on the basis of volume and structural characteristics) at the age of 4 months and 5 years (mid-point of the period up to 10 years, selected as the very early onset of puberty)	4 months: No significant differences were found between the groups fed with different methods in terms of anthropometric measurements or body composition. Among girls, no differences were found between the groups in terms of breast or uterine size. Female infants fed with MFs had a larger (*p* < 0.05) mean ovarian volume and a higher (*p* < 0.01) number of ovarian cysts per ovary than breastfed infants. 5 years: Among the females, no statistically significant differences were found between the groups fed with different methods in terms of the volume of breast buds, ovaries, or uterus; the number of ovaries with cysts; the number of ovarian cysts; the size of ovarian cysts; and the shape of the uterus.	4 months: The data obtained in the study do not confirm any significant differences related to diet in the size of reproductive organs measured by ultrasound in infants aged 4 months, although there is some evidence that ovarian development may be accelerated in MF-fed infants. SF feeding was not found to have any estrogenic effect on the reproductive organs studied in infant girls. 5 years: Among girls, no effect of early infant feeding (MF and SF) on the volume of reproductive organs and structural features in girls aged 5 years was found. Observation continues until puberty to determine the potential impact of early infant feeding on reproductive function later in life.
Duitama et al., 2018 [89] Colombia	12-month, randomized, placebo-controlled trial	Participants: *n* = 27 girls Age: 7–9 years Study group: *n* = 16 girls, fruit juice with addition of 45 g of a commercial soy protein-based supplement (SPS). Control group: *n* = 11 girls, fruit juice without addition of 45 g of a commercial soy protein-based supplement (SPS). Boys also participated in the study, but their results were not included in the article (*n* = 13 boys—study group; *n* = 11 boys—control group).	To assess the impact of soy protein supplement (SPS) consumption on sexual maturation and nutritional status in prepubertal children.	Statistically significant (*p* < 0.05) differences were observed in height and the following indices: BMI/age, weight/age, and height/age after 12 months of intervention between girls from the control and intervention groups. All girls were at stage 1 on the Tanner scale at the start of the study, and this did not change during the entire study.	Consuming SPS for 12 months did not affect sexual maturation or the onset of puberty in prepubertal girls. However, it is possible that the addition of soy protein to the diet may have caused an increase in height, BMI/age, height/age, and weight/age in girls, which was associated with changes in lean body mass.
Sinai et al., 2019 [73] Israel	Nested case-control study, based on a prospectively followed cohort	Participants: *n* = 45 girls Age: 7.8–10.5 years (mean 8.21 years), previously observed from birth until the age of 3 years. Study group: *n* = 12 girls, fed soy-based formula Control group: *n* = 33 girls, fed cow milk-based formula. Boys also participated in the study, but their results were not included in the article (*n* = 17 boys—study group; *n* = 27 boys—control group).	To investigate (prospectively) the relationship between the consumption of soy-based formulas by infants, their growth parameters, and early signs of puberty compared to cow’s milk-based formulas.	The soy group and the control group had comparable results in terms of growth and BMI z-scores. One girl in the soy group and eight girls in the control group showed early signs of puberty. No correlation was found between puberty and infant nutrition after taking into account BMI and family data. No association with puberty or differences between groups in terms of current daily intake of soy, energy, macronutrients, or micronutrients was found.	The study is the first prospective study based on physical examinations to show no association between the consumption of soy-based infant formula and growth and maturation parameters.
Felício et al., 2021 [75] Brazil	Retrospective, case-control study	Participants: *n* = 161 girls Age: Study group (mean): 7.9 ± 1.8 years; Control group (mean): 10.0 ± 2.1 years. Study group: *n* = 84 girls diagnosed with central precocious puberty. Control group: *n* = 77 without diagnosis of CPP or any symptoms of precocious puberty.	To assess the relationship between exclusive breastfeeding, soy intake and CPP.	In the control group, the percentage of children exclusively breastfed for more than 6 months was higher, which was an important protective factor against CPP (OR: 0.5; 95% CI: 0.3–0.9, *p* = 0.05). At the same time, EBF showed a negative correlation with the occurrence of CPP (r = −0.2; *p* < 0.05). Soy consumption was significantly higher in the CPP group (OR: 3.8; 95% CI: 1.5–6, *p* < 0.05) and also showed a positive correlation (r = 0.2; *p* < 0.01) with the occurrence of CPP.	The study showed that soy consumption was associated with CPP, and exclusive breastfeeding was identified as a protective factor. However, the need for further prospective studies on this topic was emphasized.
Xiong et al., 2022 [79] China	Prospective cohort study	Participants: *n* = 2152 girls Age: 6–8 years (at the beginning of study) Boys also participated in the study (*n* = 2629 boys).	To investigate whether soy consumption during childhood may prospectively influence the timing of puberty and whether dietary fiber and the key isoflavone metabolite equol may play a role in this.	Among girls, higher soy intake was associated with a statistically significant later onset of puberty (including age at Tanner stage 2 for breast development (B2) or age at the initiation of gonadal growth (G2), and age at menarche (M)), regardless of prepubertal body fat and fiber intake. These correlations were more significant among children with high urinary equol levels (*p*_for-interaction_ ≤ 0.04) or high cereal fiber intake (*p*_for-interaction_ ≤ 0.06). The consumption of dietary fiber or its subtype was not prospectively associated with the onset of puberty after adjusting for soy intake in the diet (*p* ≥ 0.06).	Higher soy intake during childhood is prospectively associated with later onset of puberty in Chinese girls. This occurs independently of pre-pubertal body fat content, and the association is particularly marked in individuals with higher urinary equol levels.

ALSPAC—the Avon Longitudinal Study of Parents and Children; AOM—age at onset of menarche; ATO—age at take-off; BF—breastfed; BMI—body mass index; CI—confidence interval; CPP—central precocious puberty; EBF—exclusive breastfeeding; HR—hazard ratio; MFs—cow milk–based formulas; OR—odds ratio; *p*—*p*-value (probability value); PHV—peak height velocity; r—correlation coefficient; SFs—soy-based formulas; SPS—soy protein-based supplement; T—tertiles, ↑ indicates an increase.

## Data Availability

No new data were created or analyzed in this study. Data sharing is not applicable to this article.
